# *Pneumocystis jiroveci* Pneumonia in HIV-Positive and HIV-Negative Patients: A Single-Center Retrospective Study

**Published:** 2019-03

**Authors:** Reem S. Almaghrabi, Sadeem Alfuraih, Rand Alohaly, Shamayel Mohammed, Abdulrahman A. Alrajhi, Ali S. Omrani

**Affiliations:** 1 Department of Medicine, King Faisal Specialist Hospital and Research Centre, Riyadh, Saudi Arabia,; 2 Department of Pathology and Laboratory Medicine, King Faisal Specialist Hospital and Research Centre, Riyadh, Saudi Arabia,; 3 Communicable Diseases Center, Hamad Medical Corporation, Doha, Qatar

**Keywords:** PJP, *Pneumocystis jiroveci*, PCP, Pneumonia, HIV, AIDS-defining condition, Trimethoprim/sulfamethoxazole, Solid organ transplant

## Abstract

**Background:**

To describe the clinical presentations, treatment regimen, and outcomes of *Pneumocystis jiroveci* pneumonia (PJP) among immunocompromised patients at King Faisal Specialist Hospital and Research Center in Saudi Arabia.

**Materials and Methods:**

In this retrospective cohort study, patients with a laboratory-confirmed diagnosis of PJP were included.

**Results:**

During the study, 42 patients with confirmed PJP were identified. Twenty (48%) patients were HIV-infected, while 22 (52%) were HIV negative. The median T-cell count (CD _4 _) was below 50 cells/mL in HIV patients with PJP at the time of HIV and PJP diagnoses. Graft rejection, cytomegalovirus (CMV) reactivation, and lymphopenia were associated with the development of PJP in transplant recipients; and high-dose steroids for non-transplant patients. The all-cause mortality at 90 days was lower in individuals with HIV-related PJP, compared to those with other predisposing conditions (10% and 32%, respectively; P=0.085). No specific risk factors were independently associated with the increased risk of mortality.

**Conclusion:**

PJP remains an important cause of morbidity and mortality in immunocompromised patients, with a higher mortality rate reported in non-HIV patients.

## INTRODUCTION

*Pneumocystis jiroveci*, formerly known as *P. carinii*, is an opportunist fungus, which may cause significant morbidity and mortality in immunocompromised patients. *Pneumocystis jiroveci* pneumonia (PJP) is one of the most commonly encountered HIV-associated opportunist infections (1). However, the increased use of combination antiretroviral therapy has resulted in a considerable decline in the incidence of PJP among HIV patients (2, 3). Other than HIV infection, patients with underlying malignancies and inflammatory conditions or those receiving steroids are also at an increased risk of PJP (4, 5). The incidence rate of PJP in solid organ transplant recipients ranges from 5% to 15%, with the highest risk reported in the first six months of transplantation and in patients receiving thoracic transplantation (6, 7).

In general, non-HIV-related PJP infections tend to present more acutely and have a more severe clinical course. They also have higher oxygen requirements and often result in mechanical ventilation (8). However, there is no evidence describing the epidemiology, presentations, or outcomes of PJP in the Kingdom of Saudi Arabia or the Middle East and North Africa. Therefore, the aim of this study was to describe the clinical characteristics, treatments, and outcomes of PJP infection in immunocompromised patients, who were managed in a single large tertiary care center in Saudi Arabia and to identify factors associated with poor clinical outcomes.

## MATERIALS AND METHODS

This retrospective cohort study was performed at King Faisal Specialist Hospital and Research Center, which is a 1200-bedded national referral center for a variety of complex medical conditions, including HIV infection, hemato-oncology, rheumatology, and organ transplant. An electronic search identified all patients with laboratory-confirmed PJP. Patients aged ≥14 years with positive silver stain for PJP in the bronchoalveolar lavage (BAL) or sputum were included from January 1, 2003 to June 30, 2016. On the other hand, patients who were diagnosed with PJP in a referral hospital were excluded.

Demographic characteristics, predisposing conditions, clinical presentations, oxygen requirements, need for mechanical ventilation, treatment, and outcomes were recorded. Indications, type, dose, and duration of immunosuppressive medications, use of PJP prophylaxis, and diagnosis of other opportunistic infections were collected, in addition to T-cell count (CD _4 _) and antiretroviral therapy details, if relevant.

### Statistical analysis

Data were summarized by measuring the percentage, median, and interquartile range (IQR), as appropriate. Chi-square and t-tests were performed to compare categorical and continuous variables, respectively. In addition, survival was compared using Kaplan-Meier curves and log-rank tests. Cox proportional-hazards model was also used to identify variables which were independently associated with all-cause mortality at 90 days. All analyses were performed using SPSS for MAC Version 21 (IBM Corporation, Armonk, New York, USA).

## RESULTS

Forty-two laboratory-confirmed PJP patients were identified and included in this study. Twenty (48%) patients were diagnosed with HIV infection, and 22 (52.4%) patients had non-HIV-related immunosuppression. The baseline characteristics, treatment, and outcomes of the patients are summarized in Table 1 and Table 2.

**Table 1.** Baseline characteristics of patients diagnosed with *Pneumocystis jiroveci* pneumonia.

**Variable**	**HIV (20)**	**Non-HIV**	**P**
Median Age in years (IQR)	42 (10.5)	41 (30)	0.366 ^†^
Male gender, number (%)	18 (90%)	11 (50%)	0.005 ^*^
Diabetes mellitus, number (%)	1(5%)	8 (36%)	0.013 ^*^
Chronic lung disease, number (%)	1 (5%)	6 (27%)	0.288 ^*^
Chronic kidney disease, number (%)	0	9 (41%)	0.001 ^*^
Chronic liver disease, number (%)	2 (10%)	1 (4.5%)	0.493 ^*^
Connective tissue disease, number (%)	0	5 (23%)	0.012 ^*^
Hematological cancer, number (%)	0	9 (41%)	0.003 ^*^
Solid organ transplant recipient, number	0	8 (36.4%)	0.101 ^*^
Median months to PJP diagnosis (IQR)	0	8 (37.75)	0.01 ^†^
Median baseline white cell count (IQR)	6.4	5.2 (3.93)	0.034 ^†^
Median baseline serum creatinine (IQR)	84 (130)	70.5 (40)	0.019 ^†^
Clinical presentation, number (%)			
Fever	14 (70%)	15 (68%)	0.899 ^*^
Cough	16 (80%)	15 (68%)	0.384 ^*^
SOB	13 (65%)	17 (77%)	0.379 ^*^
Hypoxia at presentation	9 (45%)	10 (45%)	0.976 ^*^
Requirement for mechanical	4 (20%)	4 (18%)	0.880 ^*^
Shock at presentation	1 (5%)	4 (18%)	0.188 ^*^
CXR changes, number (%)			
Airspace consolidation	14 (70%)	13 (59%)	0.461 ^*^
Interstitial infiltrate	10 (50%)	10 (46%)	0.768 ^*^
Pneumothorax	1 (5%)	2 (9%)	0.607 ^*^
Pleural effusion	3 (15%)	2 (9%)	0.555 ^*^
Concomitant CMV reactivation, number (%)	5 (25%)	9 (41%)	0.275 ^*^

IQR, interquartile range, PJP, *Pneumocystis jiroveci* pneumonia

* Chi squared,

† t-test

**Table 2.** Treatment and outcomes of patients diagnosed with *Pneumocystis jiroveci* pneumonia.

**Variable**	**HIV (20)**	**Non-HIV (22)**	**P value**
Use of IV trimethoprim-sulfamethoxazole, number (%)	11 (55%)	20 (91%)	0.098
Use of steroids for PJP therapy, number (%)	17 (85%)	18 (82%)	0.782
All-cause mortality at 30 days	1 (5%)	6 (27.3%)	0.053
All-cause mortality at 90 days	2 (10%)	7 (31.8%)	0.085

### HIV-associated PJP cohort

Twenty (48%) patients were HIV-infected. Four (20%) patients presented with PJP at the time of HIV diagnosis. In the remaining 16 patients, the median interval from HIV to PJP diagnosis was 24.5 months (IQR=90.5). The median CD _4_ count was 17 cells/mL (IQR=42) and 9.5 cells/mL (IQR=12.5) at the time of HIV diagnosis and PJP diagnosis, respectively. According to the Abbott real-time HIV assay (M2000, USA), the median baseline HIV viral load was 1.40×10^6^ RNA copies/mL (IQR=4.78×10^5^ RNA copies/mL) at the time of HIV diagnosis and 3.46×10^5^ RNA copies/mL (IQR=7.26×10^5^ RNA copies/mL) at the time of PJP presentation. The majority of patients (60%) had an HIV viral load above 10×10^6^ copies/mL at the time of PJP diagnosis.

Seven out of 20 patients (35%) had one or more simultaneous opportunistic infections at the time of PJP diagnosis, including five patients with oropharyngeal candidiasis and two patients with pulmonary tuberculosis. Twelve months before PJP diagnosis, only 5 out of 16 patients (31.3%), who had been diagnosed with HIV infection, reported PJP prophylaxis, and only three (18.8%) patients were receiving antiretroviral medications.

### Non-HIV-associated PJP

Twenty-two (22/42; 52%) patients were immunocompromised secondary to conditions other than HIV infection. Immunosuppressive conditions included solid organ transplant (8/22; 36.4%), neoplastic disorders (9/22; 40.9%), and rheumatologic diseases (5/22; 22.7%). PJP in association with solid organ transplant was diagnosed in five kidney, two liver, and one kidney-pancreas transplant recipients. The median time from diagnosis to PJP presentation was three years (IQR=2.9). Two out of eight patients developed PJP infection within the first post-transplant year following the discontinuation of PJP prophylaxis. Two patients had transplant rejection within 12 months prior to PJP diagnosis, which was treated with the addition of one or two new immunosuppressive medications. It should be noted that both patients had been restarted on trimethoprim-sulfamethoxazole for PJP prophylaxis before PJP diagnosis.

Cytomegalovirus (CMV) reactivation was documented in 4 (50%) patients, all of whom had baseline lymphopenia (median lymphocyte count= 0.45×10 ^9^ /L; IQR=0.44) (normal range= 1.5–4.3×10 ^9^ /L). Of nine patients with underlying malignant disorders, eight were diagnosed with lymphoma and one with mixed phenotype leukemia. All patients were undergoing chemotherapy at the time of PJP diagnosis. The median time from malignancy diagnosis to PJP presentation was 7.7 months (IQR=15.6), and the median time from the last chemotherapy cycle to PJP diagnosis was 27 days (IQR=77). Only three patients had received PJP prophylaxis.

Underlying rheumatologic conditions were found in three patients with systemic lupus erythematosus, two patients with systemic vasculitis, and one patient with dermatomyositis. The median dose of steroids before the diagnosis of PJP was 35 mg (IQR=73.75) for a median duration of six months (IQR=6.25). The median lymphocyte count was also low in this group of patients (0.48×19/L; IQR=0.57×10 ^9^ /L) (normal range= 1.5–4.3×10 ^9^ /L). Two patients were on other immunosuppressant medications six months before PJP diagnosis. However, none of the patients were receiving PJP prophylaxis.

**Figure 1.**
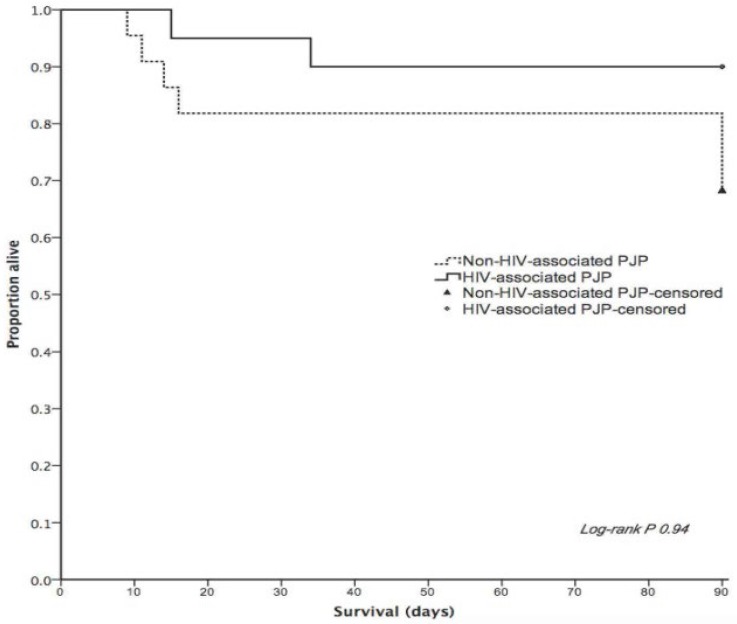
Kaplan-Meier curve of 90-day survival after PJP

The majority of our patients presented with the classic signs and symptoms of PJP in form of fever, cough, and shortness of breath, with 18–20% of patients requiring mechanical ventilation at presentation. There was no significant difference in the severity of presentation between HIV and non-HIV patients, expect for shock at presentation (higher in non-HIV cases). All patients, except two, were treated with trimethoprim-sulfamethoxazole. Nine out of 42 patients (21%) did not complete 21 days of treatment, as three of them died during therapy, one showed clinical failure, two developed renal dysfunction, one developed intractable hyperkalemia, and two developed progressive leukopenia. The overall survival was numerically higher in patients with HIV-related PJP (Figure 1).

The unadjusted Cox proportional hazard analysis for all-cause mortality at 90 days identified significant associations with the pre-exiting chronic lung disease or liver disease, shortness of breath or shock, and need for mechanical ventilation. However, no specific variables were independently associated with this outcome (Table 3).

**Table 3.** Cox proportional hazards for all-cause mortality at 90 days.

**Variable**	**Crude Hazard Ratio (95% CI)**	**P value**	**Adjusted Hazard Ratio (95% CI)**	**P value**
HIV versus non- HIV	0.11 (0.004–2.89)	0.185	0.19 (0.01–2.83)	0.205
Age	1.01 (0.97–1.06)	0.594	-	-
Male gender	1.17 (0.29–4.7)	0.820	-	-
Body weight	1.034 (0.99–1.07)	0.088	1.04 (0.99–1.10)	0.159
Smoking	0.65 (0.06–7.17)	0.724	-	-
Diabetes mellitus	1.86 (0.47–7.44)	0.381	-	-
Chronic lung disease	0.05 (0.00–4,017,636)	0.048	0.0 (0.0- -)	0.996
Connective tissue disease	2.07 (0.42–9.99)	0.364	-	-
Chronic liver disease	0.04 (0.00–1,979,634)	0.044	0.0 (0.0- -)	0.990
Chronic kidney disease	1.86 (0.47–7.44)	0.381	-	-
Solid organ transplant	1.08 (0.22–5.2)	0.924	-	-
Hematological malignancy	1.17 (0.24–5.62)	0.847	-	-
Fever	0.32 (0.09–1.18)	0.086	1.54 (0.13–18.12)	0.733
Cough	0.25 (0.07–0.92)	0.038	1.57 (0.04–64.16)	0.811
Shortness of breath	0.17 (0.04–0.68)	0.012	0.07 (0.00–1.63)	0.098
Hypoxia	1.02 (0.27–3.76)	0.981	-	-
Shock	7.04 (1.88–26.42)	0.004	1.13 (0.10–12.92)	0.919
Mechanical ventilation	3.9 (1.05–14.51)	0.043	1.9 (0.12–33.94)	0.634
CMV infection	1.69 (0.48–6.3)	0.434	-	-
Serum creatinine	1.0 (1.0–1.0)	0.034	1.0 (0.99–1.01)	0.751
Peripheral white cell count	0.97 (0.79–1.2)	0.787	-	-

CI, confidence interval

## DISCUSSION

To the best of our knowledge, this study described the largest series of PJP infection in Saudi Arabia. Despite the availability of antiretroviral medications from the late 1980’s, our patients presented with advanced HIV with the median HIV viral load >1.0×10 ^6^ RNA copies/mL and CD _4_ count <50 cells/mL. PJP was the AIDS-defining condition in 18% of the patients. This finding is consistent with the national Saudi HIV surveillance data, showing that nearly one-fifth of all new HIV diagnoses had baseline HIV viral loads above 100,000 copies/mL, besides very low CD _4_ counts (9).

In Saudi Arabia, challenges of early diagnosis and retention in care include lack of public awareness and fear of stigmatization. In a survey of more than 400 high-school students in Jeddah, the sexual route was considered as the major route of HIV transmission. In addition, kissing, handshake, and use of public bathrooms were thought as other routes of HIV transmission (10). Moreover, the findings of a cross-sectional survey of 1,483 physicians and dentists in different regions of the country are more concerning. In this survey, the participants’ general knowledge about HIV and its modes of transmission was very poor. Only 16% of the participants knew that HIV is not transmissible through saliva, and 24% correctly assumed that HIV cannot be acquired by sharing a drink with an infected person.

Additionally, stigmatizing views and attitudes towards HIV were highly prevalent, including people’s unwillingness to take care of HIV patients (40.5%), social isolation of HIV-infected patients (15%), and segregation of children with HIV from others (22.2%) (11). Overall, HIV-associated fear of stigmatization and marginalization remains a barrier to early access to care and is a major cause of poor outcomes in many parts of the world (12). In our study, the mortality rate of PJP infection in HIV patients (10%) was consistent with international studies (13, 14). CMV reactivation, graft rejection, and lymphopenia were the three main risk factors for the development of non-HIV-related PJP in our cohort study. In a recent systematic review and meta-analysis, CMV reactivation (OR=3.3; 95% CI: 2.07–5.26) and allograft rejection (OR=2.36; 95% CI: 1.54–3.62) were the most important risk factors for PJP in solid organ transplant recipients (15). Moreover, lymphopenia was previously identified as a risk factor for delayed PJP (after the first year) in solid organ transplant patients (16).

Additionally, use of steroids as part of chemotherapy or treatment of rheumatologic conditions was a major risk factor for PJP in our cohort. None of the patients with autoimmune diseases were on PJP prophylaxis despite receiving steroids for a median duration of six months. However, the role of PJP prophylaxis in patients on chronic steroids remains controversial. While some reports supported our findings (17, 18), others concluded that this association needs stronger evidence (19). In this regard, one prospective, multi-center trial found that 80% of patients with PJP were on prolonged steroids (20).

At present, there is no consensus as to whether PJP prophylaxis should be routinely incorporated in the care of patients with rheumatologic conditions, who are expected to be on steroids for prolonged periods. In our cohort, this group of patients had low absolute lymphocyte counts of 0.48 10 ^9^ /L (normal: 1.5–4.3 10 ^9^ /L), which might have increased the risk of PJP infection, besides prolonged steroids. There was no significant difference in the presentation or severity of PJP between HIV and non-HIV patients in our cohort, but there was a clear trend towards poor outcomes and higher mortality in non-HIV patients.

Although this retrospective study was conducted in a single center, it is the largest report from the Middle East and North Africa, including all patients at risk of PJP. Therefore, a prospective multi-center study is required to confirm our findings.
